# Opsin expression predicts male nuptial color in threespine stickleback

**DOI:** 10.1002/ece3.4231

**Published:** 2018-06-11

**Authors:** Chad D. Brock, Diana Rennison, Thor Veen, Daniel I. Bolnick

**Affiliations:** ^1^ Department of Integrative Biology University of Texas at Austin Texas; ^2^ Biodiversity Institute & the Department of Botany University of Wyoming Laramie Wyoming; ^3^ Institute of Ecology and Evolution University of Bern Bern Switzerland; ^4^ Life Sciences Quest University Squamish BC Canada

**Keywords:** gene expression, nuptial color, opsins, sexual selection

## Abstract

Theoretical models of sexual selection suggest that male courtship signals can evolve through the build‐up of genetic correlations between the male signal and female preference. When preference is mediated via increased sensitivity of the signal characteristics, correlations between male signal and perception/sensitivity are expected. When signal expression is limited to males, we would expect to find signal‐sensitivity correlations in males. Here, we document such a correlation within a breeding population of threespine stickleback mediated by differences in opsin expression. Males with redder nuptial coloration express more long‐wavelength‐sensitive (LWS) opsin, making them more sensitive to orange and red. This correlation is not an artifact of shared tuning to the optical microhabitat. Such correlations are an essential feature of many models of sexual selection, and our results highlight the potential importance of opsin expression variation as a substrate for signal‐preference evolution. Finally, these results suggest a potential sensory mechanism that could drive negative frequency‐dependent selection via male–male competition and thus maintain variation in male nuptial color.

## 1 INTRODUCTION

Animals have evolved phenomenally diverse courtship traits, inspiring numerous theoretical models to explain their evolution (Darwin, 1871; Fisher, 1930; Lande, 1981; Kirkpatrick, 1987; Kirkpatrick & Ryan, 1991; Andersson, 1994; Kirkpatrick, 1996; Arnqvist & Rowe, 2005; Fuller et al. 2005; Andersson & Simmons, 2006; Kokko, Jennions, & Brooks, 2006; Ryan & Cummings, 2013) and empirical tests of these models (Andersson, 1994; Arnqvist & Rowe, 2005; Cummings, 2007; Kirkpatrick, 1987; Kirkpatrick & Ryan, 1991; Kokko et al., 2006; Ryan & Cummings, 2013). A common feature of these theoretical models entails an increasing genetic correlation between a courtship signal and the signal preference, resulting in their correlated evolution (Fisher, 1930; Kirkpatrick, 1996; Kirkpatrick & Ryan, 1991; Lande, 1981). For example, in “good genes” models of sexual selection, nonrandom associations between a courtship trait and genes underlying good condition can select for alleles conferring preference for mates displaying so‐called indicator traits reflecting high quality. Consequently, a genetic correlation arises between female preference for the male trait, the male signal, and the genes conferring higher fitness. These genes then jointly increase in frequency due to natural selection. The resulting increase in frequency of female preference alleles produces stronger sexual selection on the courtship trait, further increasing the frequency of both the trait and preference (the “runaway” process; Fisher, 1930; Lande, 1981).

Previous work has reported genetic correlations between courtship signals and preferences in a number of species (Bakker, 1993; Houde, 1994; Limousin et al., 2012; Rick, Mehlis, & Bakker, 2011; Wiley & Shaw, 2010; Wilkinson & Reillo, 1994). For example, Bakker (1993) bred wild‐caught threespine stickleback to reveal a genetic correlation between female preference for male red throat coloration and male red throat intensity. This association is partially mediated through an increased visual sensitivity to red in female offspring of redder males (Rick et al., 2011). However, the mechanism(s) responsible for this association between a males’ color and their female offspring's visual sensitivity and preference remains unclear. One possibility is that male color may be genetically correlated with opsin expression, both of which vary within stickleback populations (Reimchen 1989; Boughman, 2001; Rennison, Owens, Heckman, Schluter, & Veen, 2016; Brock, Bolnick, & Cummings, 2017a; Veen, Brock, Rennison, & Bolnick, 2017), are heritable (Lewandowski & Boughman, 2008; Rennison et al., 2016; Malek, Boughman, Dworkin, & Peichel, 2012; but see Brock et al., 2017a; Brock, Bolnick, & Cummings, 2017b; Veen et al., 2017) and have been shown to covary with color in other systems (Bloch, 2015; Price, 2017; Sandkam, Young, & Breden, 2015). We would then expect that signal and visual physiology would also be correlated within the males themselves. That is, redder male stickleback might express proportionately more of the orange‐red‐sensitive LWS opsin. Both traits may be inherited (genetically or otherwise) by all offspring, but only in male offspring (which alone express the signal) could we detect a signal‐sensory correlation. Consequently, opsin expression variation would mediate covariance between the male signaling trait and female trait preference and serve as an important substrate for signal‐preference evolution.

Within‐individual correlations between signal and sensitivity could also influence male–male competition. For example, positive covariance between red coloration and sensitivity to red in males could drive greater antagonism between red males, leading to negative frequency‐dependent selection that could favor rare male color morphs and thus maintain variation in nuptial color (Djikstra, Seehausen, Gricar, Maan, & Groothuis, 2006; Djikstra, Seehausen, Pierotti, & Groothuis, 2006; Seehausen & Schluter, 2004; Bolnick et al. 2016; Djikstra & Border 2018). Bolnick et al. (2016) provided evidence for negative frequency‐dependent selection via male–male competition in two lake populations of stickleback that differ in male nuptial color. In lakes where native males are predominantly red‐throated, red‐throated models were attacked more frequently and with a higher intensity, while the opposite was the case for lakes with melanic males (Bolnick et al. 2016; but see Tinghitella, Lehto, & Minter, 2015). These results were also modulated within lakes by depth, a proxy for optical environment (Bolnick et al. 2016; Brock et al., 2017a; Brock, Bolnick, & Cummings, 2017b). Threespine stickleback males display color polymorphism at multiple geographic scales (Reimchen 1989; Boughman, 2001; Scott, 2001; Brock et al., 2017a; Brock, Bolnick, & Cummings, 2017b; Marques, Lucek et al., 2017; Marques, Taylor et al., 2017). As these color phenotypes typically covary with features of the optical environment, sensory drive theory is commonly invoked to explain the maintenance of multiple color phenotypes (Reimchen 1989; Boughman, 2001; Scott, 2001; Brock et al., 2017a; Brock, Bolnick, & Cummings, 2017b; Marques, Lucek et al., 2017; Marques, Taylor et al., 2017). However, negative frequency‐dependent selection via male–male competition could also facilitate the maintenance of color polymorphisms, possibly via interactions with the signaling environment (Bolnick et al. 2016; Djikstra & Border, 2018; Tinghitella et al., 2017).

Here, we document covariance between male color and male opsin expression within a single population of breeding threespine stickleback males. Specifically, we find that redder males express more of the long‐wavelength‐sensitive (LWS) opsin, and visual modeling suggests this increased expression leads to greater sensitivity to orange and red. Furthermore, visual modeling results and path analyses suggest that this correlation is not an artifact of shared tuning to the optical microhabitat, which varies with nest depth. These results provide further evidence for signal‐preference/sensitivity correlations, as well as highlight the potential importance of opsin expression variation as a substrate for both female preference evolution (Bakker, 1993; Bloch, 2015, 2016; Lind, Henze, & Osorio, 2017; Price, 2017; Rick et al., 2011; Sandkam et al., 2015) and negative frequency‐dependent selection via male–male competition (Bolnick et al. 2016).

## 2 MATERIALS

### 2.1 Male collection and reflectance measurements

In 2014, snorkelers used dipnets to capture nesting male stickleback (*n* = 16) from Gosling Lake on Vancouver Island, British Columbia. This is the same red‐throated lake population used in Bolnick et al. (2016). We recorded each males’ nest depth prior to capture. We sampled nesting males over three consecutive days to minimize temporal variance in male color. A darkened cooler with fresh lake water was used to transport males to shore for immediate collection of reflectance data (within 1–5 min after capture). This cooler was rinsed and refilled with lake water between males. For each male, spectral reflectance measurements were taken using an EPP200C UV‐VIS spectrometer, SL‐4 Xenon lamp, and a R400‐7 reflectance probe for two body regions: 1) preoperculum and 2) abdomen. Reflectance data were collected from live, unanesthetized males suspended in an aquarium constructed by CDB with UV‐transmissive material. All measurements were taken while holding the probe flush with the side of the aquarium and perpendicular to the surface of the fish. Three replicate measurements were collected for each body region, removing and reapplying the probe between each replicate. Spectralon white standard measurements were taken between each fish to account for lamp drift. The aquarium was emptied, rinsed, and refilled with fresh lake water between males. For our color metric, we calculated the ratio of the respective areas under the reflectance curve for the wavelength intervals of 301–400 nm (UV‐blue), 401–500 nm (blue‐green), 501–600 (green‐orange), and 601–700 (orange‐red) for each body region. These four color proportions represent the raw color data we use for our analyses.

### 2.2 Opsin expression

Stickleback have four cone opsin genes: short‐wavelength‐sensitive 1 (SWS1: $\lambda_{\max}$ = 365–382 nm); short‐wavelength‐sensitive 2 (SWS2: $\lambda_{\max}$ = 434–441 nm); middle‐wavelength‐sensitive (RH2: $\lambda_{\max}$ = 514–546 nm); and long‐wavelength‐sensitive (LWS: $\lambda_{\max}$ = 566–638 nm) (Brock et al., 2017a; Flamarique, Cheng, Bergstrom, & Reimchen, 2013; McClennan, 2007; Rennison et al., 2016; Rowe, Baube, Loew, & Phillips, 2004; Veen et al., 2017). We measured the relative abundance of mRNAs for each of these four opsin genes for all 16 males, as described in Veen et al. (2017). Total RNA was extracted from a mixed homogenate from both left and right eyes and used to synthesize cDNA for RT‐qPCR analysis. For each male, we summed the opsin gene expression across the four cone opsin genes and estimated the proportion of total expression for each gene. This provides a measure of relative gene expression.

### 2.3 Irradiance

We measured sidewelling and downwelling irradiance along a depth gradient (0–2.5 m) at 50‐cm intervals. Irradiance data were collected with an EPP200C UV‐VIS spectrometer linked to a UV‐NIR cosine receptor. Sidewelling irradiance was collected with the probe oriented horizontally toward the shore and represents the optical background against which a male stickleback is often viewed by conspecifics. Downwelling irradiance was measured with the probe directed vertically toward the water surface and represents the primary source of light for target reflection. Irradiance measurements (W/m^2^) were converted into μE m^−2^ s^−1^ using a LI‐COR Optical Radiation Calibrator (model 1800‐02) calibration lamp. Three replicate measurements were taken at each depth, and these measurements were dispersed throughout the nesting environment. We used the median value at each wavelength of these three replicates as our estimate of the ambient light at a given depth. As both the time of day and the time of year can impact irradiance measurements, all irradiance data were collected between 9 and 10:30 a.m. on a single day. To control for ambient light conditions, we collected measurements immediately above and below the water surface for each replicate to allow for the normalization of irradiance (Normalized Irradiance_Depth_ = Irradiance_Depth_/Irradiance_Surface_). As results were not impacted by irradiance normalization, we focus on the results using the raw (=non‐normalized) irradiance data in the paper. A more detailed discussion of the collection of irradiance data is given in Veen et al. (2017).

### 2.4 Stickleback visual model

To assess whether opsin expression differences influenced visual sensitivity we employed a stickleback visual model developed in Brock et al. (2017a). Specifically, we developed a photoreceptor noise‐limited color discrimination model for stickleback fish using MSP‐estimated peak cone sensitivities and employed the parameters outlined in reference Govardovskii, Fyhrquist, Reuter, Kuzmin, and Donner (2000) to calculate spectral sensitivity functions for the four stickleback cone receptors. The absolute quantum catch, *Q*, for each class of photoreceptor:

$$Q_{c}=\int_{\lambda =300}^{700}A_{c}\left(\lambda \right)S\left(\lambda \right)I\left(\lambda \right)d\lambda ,$$

where λ is the wavelength, *A*_*c*_ is the photoreceptor absorptance of cone class *c*,* S(*λ*)* is the target reflectance, and *I(*λ*)* is the environment irradiance. Quantum catch was adjusted for the adapting light environment using von Kries transformations, so that *q*_*c*_ *= k*_*c*_ *Q*_*c*_, and

$$k_{c}=\frac{1}{\int_{\lambda =300}^{700}A_{c}\left(\lambda \right)I_{b}\left(\lambda \right)d\lambda }$$

where *I*_*b*_ *(*λ*)* is the adapting background. The signal for each photoreceptor when viewing a target in a given background is proportional to the logarithm of their adjusted quantum catches such that contrast between the two is

$$\Delta f_{c}=\ln\frac{q_{c}\left(\text{target}\right)}{q_{c}\left(\text{background}\right)}$$

Threespine stickleback are tetrachromats and thus have four cone classes: UV‐sensitive (SWS1, abbreviated below as U), short‐wavelength‐sensitive (SWS2 = S), medium‐wavelength‐sensitive (MWS = M), and long‐wavelength‐sensitive (LWS = L) cone receptors. As such, the perceptual distance in terms of chromatic contrast, *∆S*, between the target and background was calculated for a tetrachromatic visual system as

$$\Delta S^{2}=\frac{\left[\begin{array}{cc} & {\left(e_{1}e_{2}\right)}^{2}{\left(\Delta f_{4}-\Delta f_{3}\right)}^{2}+{\left(e_{1}e_{3}\right)}^{2}{\left(\Delta f_{4}-\Delta f_{2}\right)}^{2}+\\ & {\left(e_{1}e_{4}\right)}^{2}{\left(\Delta f_{2}-\Delta f_{3}\right)}^{2}+{\left(e_{2}e_{3}\right)}^{2}{\left(\Delta f_{4}-\Delta f_{1}\right)}^{2}+\\ & {\left(e_{2}e_{4}\right)}^{2}{\left(\Delta f_{3}-\Delta f_{1}\right)}^{2}{\left(e_{3}e_{4}\right)}^{2}{\left(\Delta f_{2}-\Delta f_{1}\right)}^{2} \end{array}\right]}{\left[{\left(e_{1}e_{2}e_{3}\right)}^{2}+{\left(e_{1}e_{2}e_{4}\right)}^{2}+{\left(e_{1}e_{3}e_{4}\right)}^{2}+{\left(e_{2}e_{3}e_{4}\right)}^{2}\right]}$$

where *e*_*c*_ is the signaling noise for a photoreceptor of class *c* and is given by the following:

$$e_{c}=\sqrt{\frac{\omega }{\eta_{c}}}$$

where *ω* is the Weber fraction, and *η*_c_ is relative density of photoreceptors of class *c* in the retina. A Weber fraction value of 0.05 was chosen as a conservative estimate.

Finally, it is advisable to incorporate the transmission spectra of the lens, ocular media, and intracone oil droplets (if present) when calculating target contrasts using visual models, as these can strongly influence spectral sensitivity in vertebrates, including fish (Bowmaker, 2008; Bowmaker, Heath, Wilkie, & Hunt, 1997; Douglas & Jeffrey, 2014; Lind, Mitkus, Olsson, & Kelber, 2014; Loew, Fleishman, Foster, & Provencio, 2002; Stavenga & Wilts, 2014). However, to our knowledge, there are no available transmission data for the lens or ocular media of threespine stickleback (Rick, Bloemker, & Bakker, 2012 measure lens transmission, but their data are not publicly available; see also Flamarique, Bergstrom, Cheng, & Reimchen, 2013). Similarly, it is unknown whether oil droplets are present within the cones of threespine stickleback. As such, we were not able to include any of these components in our visual model calculations. While we recognize this is a limitation of our study, previous evidence from both optomotor and behavioral studies indicate that stickleback are sensitive to a variety of colors, including those of both long (e.g., red) and short (e.g., ultraviolet) wavelengths (e.g., Rick & Bakker, 2008a,b,c; Flamarique, Bergstrom et al., 2013; Flamarique, Cheng et al., 2013; Shao et al., 2014). Consequently, these results strongly suggest the stickleback eye is at least partially transmissive to wavelengths between ~300 and 700 nm. Additionally, the lens of a close relative, the fifteen‐spine stickleback (*Spinachia spinachia*), is highly transmissive across the visible spectrum, including UV (Thorpe et al. 1993), which is consistent with the presence of similar lens properties in the threespine stickleback. Given these previous results, we feel the conclusions of our visual model analyses should be qualitatively robust to the exclusion of these components from our calculations of the quantum catch.

We combined our opsin expression estimates with this visual model to calculate normalized absorbance across the visible spectrum. Specifically, we adjusted the relative density of the photoreceptors in the retina (*η*_c_) in our model to match our opsin expression profiles for each male. As the range of LWS peak sensitivity (see above) may vary based on the nature of the bound chromophore (11‐*cis* retinal vs. 11‐*cis* 3,4 didehydroretinal, often designated by their precursor vitamins A_1_ and A_2_; Flamarique, Bergstrom et al., 2013; Flamarique, Cheng et al., 2013; Enright et al., 2015), we conducted the analyses using the lower bound (566 nm), upper bound (638 nm), and the median (i.e., essentially averaging across chromophore types in the retina; see Rennison et al., 2016 & Veen et al., 2017). From this, we then estimated visual sensitivity to orange‐red (i.e., summed absorbance between 590 and 650 nm) and tested whether this estimate of orange‐red sensitivity covaries with male coloration. Additionally, we employed this visual model to calculate chromatic contrast, *ΔS* (see above), to assess whether male color and opsin expression are matched to their local optical microhabitat. Specifically, we permuted opsin expression and color across males, and then calculated contrast for all opsin‐color pairings at each nesting depth (i.e., an in silico transplant experiment). This allowed us to calculate each male's contrast, as viewed by every possible opsin phenotype against every possible visual background. The average across all opsin‐color pairings for a given nesting depth provides a null expectation for contrast. We then calculated each male's observed contrast (using its own color and opsin profile at its actual nest depth). Next, we calculated each males’ deviation from the null expectation (*ΔS*_empirical_–*ΔS*_null_). If males were more or less conspicuous than the null, this would suggest a nonrandom association between color, opsins, and optical microenvironment.

### 2.5 Statistical analyses

To test for an association between color and opsin expression, we used the “*CCA”* package (González & Déjean, 2012) in R to conduct canonical correlation analyses. We examined the preoperculum and abdomen separately, and estimated canonical correlations between the four color proportions (see *Male collection and reflectance measurements*) and the relative expression levels of the four stickleback opsins. The significance of the canonical axes was assessed sequentially with permutation tests and Wilk's lambda as a test statistic, using the “*CCP”* package in R (Menzel, 2012). To test whether red males showed evidence for increased sensitivity to long wavelengths (orange‐red wavelengths, 590–650 nm), we used linear regression of male color vs. long‐wavelength absorbance.

To investigate whether empirical color‐opsin pairings were nonrandomly associated with nest depth, we conducted a one‐sample Student's t test of the deviation of a male's empirical contrast from the null expectation at the male's nest depth (*ΔS*_empirical_–*ΔS*_null_). Additionally, to account for the potential confounding effect of nest depth (Brock et al., 2017a; Brock, Bolnick, & Cummings, 2017b; Veen et al., 2017), we conducted a path analysis using the “*sem”* package (Fox, Nie, & Byrnes, 2017) in R. We calculated the direct correlation between color and opsins (using the first canonical variate scores for each data matrix) while accounting for their possible shared correlation with nest depth.

## 3 **RESULTS**

**Figure 1:**
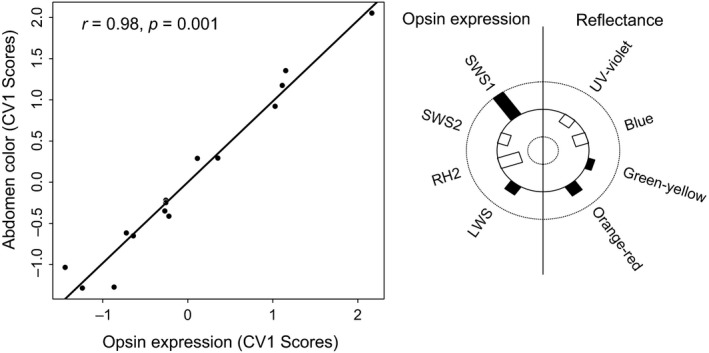
Males that reflect more red display higher relative expression of the red‐sensitive LWS opsin. (a) Plot of the first canonical variate for color and opsin expression and the first canonical correlation between these variates for the abdomen. (b) Helioplot showing the canonical loadings along the first canonical variate axes for male reflectance and opsin expression. Solid bars indicate positive loadings, and clear bars indicate negative loadings

**Figure 2:**
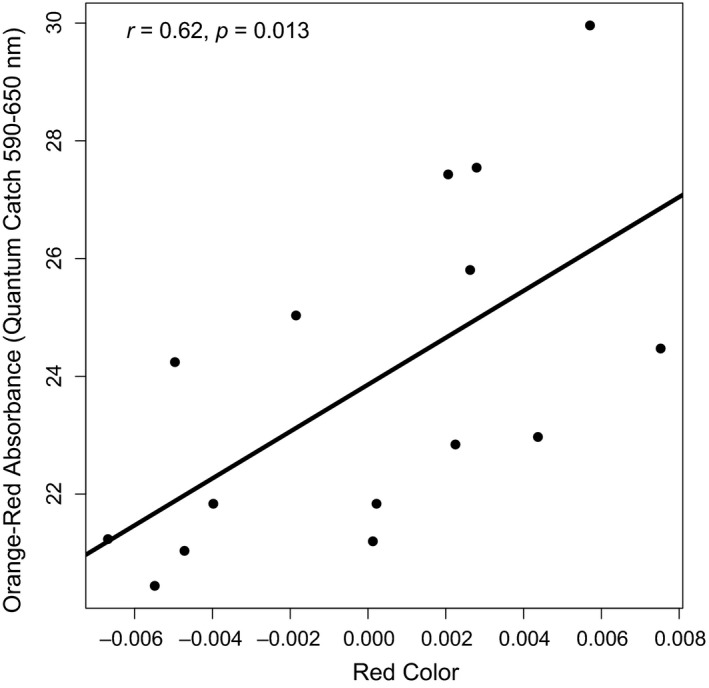
Redder males show greater absorbance (quantum catch) in the retina of orange‐red wavelengths due to their higher expression of the LWS opsin. Orange‐red (590–650 nm) absorbance is significantly correlated with the intensity of red coloration within males. The results displayed here were calculated using the median $\lambda_{\max}$ that averages across chromophores

The CCA confirmed a positive correlation between redder coloration (of both body regions) and relative expression of the long‐wavelength‐sensitive LWS opsin (Figure 1). Permutation tests indicate that the first canonical correlation was significant for the abdomen alone (canonical correlation_1_; *r* ^2 ^= 0.982, *p* = 0.001; Figure 1). For this same axis, males with more UV‐reflective abdomens expressed relatively less of UV‐sensitive SWS1 opsin (Figure 1b). Visual model analysis indicated that redder males had higher absorbance in orange‐red range of the visible spectrum, suggesting an increased sensitivity to long wavelengths in these individuals (*r* ^2^=0.38, *p* = 0.013; Figure 2). These results were consistent across the different LWS $\lambda_{\max}$ inputs, so we focus on the results using the median $\lambda_{\max}$ that averages across chromophores (Figure 2).

Path analyses indicated that these signal‐sensory correlations are not due to a shared correlation with depth, instead supporting a direct association that is significant for both the abdomen and preoperculum (Table 1). Likewise, the in silico “transplant experiment” indicated that at a given nest depth the resident males were not more visible, nor better able to see other males, compared with randomly transplanted males from other depths (preoperculum: *t*_*15*_ = −0.414, *p* = 0.684; abdomen: *t*_*15*_=−0.864, *p* = 0.402). As depth is strongly correlated with the ambient light environment (Brock et al., 2017a,b; Veen et al., 2017), these results suggest that color‐opsin covariance is not an incidental result of each trait's separate tuning to the local optical regime.

**Table 1:** Path analyses provide evidence for a direct association between male color and opsin expression for both the abdomen and the preoperculum. Bold values indicate statistically significant associations (*p* < 0.05)

Preoperculum	Estimate	*SE*	z‐value	*p*‐value
Depth → Opsins	0.423	0.437	0.967	0.333
Depth → Color	0.097	0.451	0.215	0.829
Opsins ↔ Color	0.892*	0.351	2.537	***0.011***

Abdomen	Estimate	*SE*	z‐value	*p*‐value
Depth → Opsins	−0.866*	0.388	−2.232	***0.026***
Depth → Color	−0.706	0.411	−1.721	0.085
Opsins ↔ Color	0.768*	0.293	2.625	***0.008***

## 4 DISCUSSION

We found that within a given male stickleback nuptial color was strongly correlated with opsin expression. Specifically, redder males had elevated expression levels of long‐wavelength‐sensitive LWS opsin. Our visual modeling results suggest these redder males are more sensitive to orange‐red. Higher UV‐reflecting males expressed relatively less UV‐sensitive SWS1. Despite previous studies finding correlations between nest depth and both male color and opsin expression (Brock et al., 2017a,b; Veen et al., 2017), in the present sample we found an effect of depth on opsins alone. Our visual model also provided no evidence that color and opsin expression were jointly tuned to the local optical microhabitat.

Our results suggest a plausible mechanism for the previously reported genetic correlation between male sticklebacks’ red color, and their daughters’ sensitivity to and preference for red mates (Rick et al., 2011). Stickleback nuptial color and opsin expression are both reported to be heritable (Lewandowski & Boughman, 2008; Malek et al., 2012; Rennison et al., 2016) and evidence suggests the latter is not sex‐biased (Veen et al., 2017). Thus, it is reasonable to hypothesize that redder males with more LWS expression should tend to have female offspring who also express relatively more LWS opsin. Consequently, these females may be more prone to detect and choose red mates (Bakker, 1993; Rick et al., 2011), potentially increasing genetic covariance between signal and preference. However, as we did not directly assess the trait heritability in this focal population, nor have we yet directly tested for effects on female preference, this remains a hypothesis in need of further investigation. Consistent with this hypothesis, recent work in stickleback and cichlids suggests relative LWS opsin(s) expression influences optomotor thresholds (Shao et al., 2014; Smith, Ma, Soares, & Carleton, 2012) and correlates with mate preference across populations of guppies (Sandkam et al., 2015), and future assays in stickleback could assess whether opsin expression predicts visual sensitivity, as our visual modeling suggests, and mate preference. Additionally, future studies can employ breeding experiments to further assess the heritability of color and opsin expression profiles (i.e., relative proportions of each opsin expressed) in offspring of both sexes (sensu Flamarique, Bergstrom et al., 2013; Flamarique, Cheng et al., 2013; Rennison et al., 2016), as well as directly measure genetic correlations between these traits (sensu Bakker, 1993). Finally, as diet is one potential mechanism for mediating covariance between color and opsin expression (see below), it is worth highlighting that parent–offspring correlations between color and opsin expression could result from less direct genetic (i.e., heritability in diet or trophic morphology influencing rate of carotenoid intake) or nongenetic mechanisms (learned dietary preferences).

Color‐opsin correlations within individual males could also influence male–male competition and may help explain previous evidence for depth‐mediated negative frequency‐dependent selection in stickleback (Bolnick et al. 2016). Negative frequency‐dependent selection favors rare phenotypes, thus maintaining phenotypic variation within populations (Bolnick, 2004; Seehausen & Schluter, 2004; Kopp & Hermisson, 2006; Nosil, 2006; Olendorf et al., 2006; Rueffler, VanDooren, Leimar, & Abrams, 2006; Djikstra, Seehausen, Gricar, et al., 2006; Djikstra, Seehausen, Pierotti, et al., 2006; Bolnick et al. 2016; Djikstra & Border, 2018), and previous empirical work suggests negative frequency‐dependent selection can maintain male color polymorphism in natural populations (Seehausen & Schluter, 2004; Nosil, 2006; Djikstra, Seehausen, Gricar, et al., 2006; Djikstra, Seehausen, Pierotti, et al., 2006; Bolnick et al. 2016; But see Gray et al., 2008).

Bolnick et al. (2016) demonstrated that male–male antagonistic interactions were frequency‐dependent among lake populations of threespine stickleback (Gosling Lake and Blackwater Lake). Gosling Lake males, which are typically red‐throated, attacked red‐throated models more often and with a greater intensity than melanic models, while the opposite pattern was the case for melanic males in Blackwater Lake. These results were also modulated by depth, a proxy for optical environment, which can influence male signal contrast to a specific perceiver, that is, a male competitor (reviewed in Ryan & Cummings, 2013; Brock et al., 2017a). Red‐throated males attacked red models even more frequently in shallow environments, while melanic males attacked melanic models more frequently in deep environments. Covariance between color and opsin expression within individuals males may provide a viable mechanism to explain these patterns. Lakes with red males should, on average, have higher levels of LWS expression, increasing their sensitivity to redder males. Consequently, this increased sensitivity could drive more frequent and intense aggression toward redder males. Furthermore, opsin expression profiles show a significant depth gradient in Gosling Lake male stickleback (Table 1; Veen et al., 2017), with higher LWS expression in shallow environments (Table 1; Figure 1b; Veen et al., 2017). As our visual modeling results suggest, shallow males with higher LWS expression would have elevated sensitivity to orange‐red reflectance, and this again could drive greater aggression toward red males. While this pattern could reflect elevated contrast of redder males, our visual modeling analyses suggest that local males were not more conspicuous in their home environments when viewed by a local perceiver. Thus, redder males were not more conspicuous in shallow environments to local males (i.e., those with elevated expression of LWS). These results are consistent with previous work in Gosling Lake that found red‐throated males were actually less conspicuous than null expectations in their native shallow environments, suggesting that something other than (or in addition to) signal contrast is driving depth gradients in male color (Brock et al., 2017a). Thus, while covariance between color and opsins is a potential mechanism that could drive negative frequency dependence of male–male antagonism at both geographic scales, it seems unlikely that this is mediated through increased conspicuousness alone. Future work could replicate the study design of Bolnick et al. (2016) while additionally collecting opsin data from focal males to assess whether opsin profiles 1) covary with male color between lakes and 2) predict male antagonism to color models.

The proximate mechanism responsible for this color‐opsin correlation remains unknown. One possibility entails variance in carotenoid consumption and/or allocation. The red coloration of breeding male stickleback is partly based on diet‐derived carotenoids (Pike, Blount, Lindstrom, & Metcalfe, 2010; Wedekind, Meyer, Frischknecht, Niggli, & Pfander, 1998). Carotenoids may also influence opsin expression, and recent experimental work in guppies demonstrated that increased consumption of carotenoids led to increased expression of LWS opsins (Sandkam et al., 2016). Thus, diet variation among individuals (heritable *or* learned) (Snowberg & Bolnick, 2012) could lead to variation in carotenoid availability that jointly affects male color and opsin expression. While future work is needed to investigate the mechanism and genetics of this color‐vision correlation, our results present a clear example of covariance between nuptial signal and the capacity to perceive that signal. Trait correlations such as these are an essential feature of many models of sexual selection and signal evolution and can help explain the apparent concerted evolution of signal and preference in nature. Our results highlight the potential importance of opsin expression variation as a substrate for female preference evolution (Bloch, 2015, 2016; Lind et al., 2017; Price, 2017; Sandkam et al., 2015). Finally, these results suggest a potential sensory mechanism that could facilitate the maintenance of variation in male nuptial color via negative frequency‐dependent selection (Bolnick et al. 2016).

## CONFLICT OF INTEREST

None declared.

## AUTHOR CONTRIBUTIONS

All authors designed the study and contributed to article revisions. CDB, DJR, and TV collected the data. CDB did the majority of the analyses and wrote the manuscript. All authors agree to be held accountable for the contents of this work and approve the final version of the manuscript.

## ETHICS

Specimens were collected with permission of the Ministry of Environment of British Columbia (permit NA14‐93580) and the University of Texas IACUC (AUP‐2012‐00098).

## DATA ACCESSIBILITY

Data will be posted on Dryad.org at the time of publication.
